# How do (false) positively screened patients experience a screening programme for liver cirrhosis or fibrosis in Germany? A qualitative study

**DOI:** 10.1111/hex.13800

**Published:** 2023-06-19

**Authors:** Urs A. Fichtner, Anita Arslanow, Harald Binder, Peter R. Galle, Christian Labenz, Frank Lammert, Julia Ortner, Dominikus Stelzer, Louis Velthuis, Erik Farin‐Glattacker

**Affiliations:** ^1^ Section of Health Care Research and Rehabilitation Research, Institute of Medical Biometry and Statistics, Faculty of Medicine and Medical Center University of Freiburg Freiburg Germany; ^2^ Department of Internal Medicine I University Medical Center of the Johannes Gutenberg‐University Mainz Germany; ^3^ Institute of Medical Biometry and Statistics, Faculty of Medicine and Medical Center University of Freiburg Freiburg Germany; ^4^ Institute for Occupational Medicine and Public Health Saarland University Homburg Germany; ^5^ Hannover Medical School Hannover Germany; ^6^ Department of Law and Economics Johannes Gutenberg University Mainz Germany

**Keywords:** cirrhosis, fibrosis, liver screening, patient‐reported outcomes, psychosocial consequences

## Abstract

**Objective:**

This study aimed to explore psychosocial consequences of (false) positive liver screening results and to identify influencing factors for perceived strain within a multistage screening programme for liver cirrhosis and fibrosis in Germany.

**Methods:**

Between June 2018 and May 2019, all positively screened patients were asked to participate in the study (*n* = 158). *N* = 11 telephone interviews and *n* = 4 follow‐up interviews were conducted. Semi‐structured telephone interviews were carried out. The analysis followed a structuring content analysis approach. Thereby, categories were first defined deductively. Second, the categories were revised inductively based on the data.

**Results:**

The main themes found regarding the consequences of the screening were categorised in emotional reactions and behavioural reactions. Few respondents described negative emotional consequences related to screening. Those seem to be mostly driven by suboptimal patient–provider communication and might be worsened when transparent information transfer fails to happen. As a result, patients sought information and support in their social environment. All patients reported positive attitudes towards liver screening.

**Conclusion:**

To reduce the potential occurrence of psychosocial consequences during the screening process, medical screening should be performed in the context of transparent information. Regular health communication on the side of health professionals and increasing patients' health literacy might contribute to avoiding negative emotions in line with screening.

**Patient or Public Contribution:**

This study recognises the wide‐ranging patients' perspectives regarding the consequences of liver screening which should be taken into consideration when implementing a new screening programme to ensure a patient‐centred approach.

## 1 INTRODUCTION

Medical screening aims at identifying diseases in their preclinical phase to prevent severe progression.^1^ Typically, screening is used to detect diseases before symptoms are present and thus, before patients seek medical advice for a specific problem.^2^ Therefore, screening has the potential to move patients from a state of supposing themselves as healthy to the state of having a medical disorder. Reception of a positive test result represents a stress factor and can have severe psychosocial consequences.^3^ This is especially an ethical problem, if screening results are false positive.^4^, ^5^, ^6^, ^7^, ^8^ Besides the benefits of early detection of diseases, such as early treatment and potential prevention of progression, those negative effects should also be taken into consideration when evaluating new forms of screenings.^9^, ^10^ It is known from various studies on different screenings for cancer, that (false positive) abnormal findings in screening can lead to sleeping disorders,^4^ increased anxiety,^4^, ^11^ psychological distress,^12^, ^13^ sadness,^12^ restlessness,^12^ fears^14^ and considerations on future participation in screening.^15^ However, those consequences do not occur consistently. It is important to differentiate between long‐term and short‐term effects^5^, ^13^ as well as between disease‐specific and general outcomes.^8^

In addition to false‐positive results, intensive surveillance during the screening process itself can produce unfavourable side effects on psychological well‐being and health‐related quality of life due to the confrontation with a potential threat.^16^

The majority of studies reporting on psychosocial effects of screening refer to different types of cancer screening programmes such as breast,^8^ colorectal,^17^ anal^18^ and skin cancer.^19^ Little is known about the impact of other medical screenings, for example, for advanced liver fibrosis or cirrhosis.

Cirrhosis is the common end stage of a chronic liver disease that often develops unnoticed over the years and thus is most often diagnosed in a late phase when complications occur.^20^ In many cases, the transition between advanced liver fibrosis and cirrhosis is fluid. In this stage, causative treatment is less successful or impossible.^21^ Furthermore, it constitutes a risk factor for the onset of liver cancer. Even in highly developed health care systems, cirrhosis is diagnosed in an asymptomatic early stage only in about 25% of patients.^22^

In January 2018, the SEAL programme (structural early‐detection of asymptomatic liver cirrhosis or fibrosis) was implemented for 39 months in two German federal states (Rhineland‐Palatinate and Saarland) aiming to investigate the feasibility, effectiveness and cost‐effectiveness of a general screening programme for liver fibrosis and cirrhosis in primary care.^21^ Within this programme, patients who were members of the statutory health insurance (Allgemeine Ortskrankenkasse Rhineland‐Palatinate/Saarland—AOK) and who were eligible for participation (inclusion criteria: signed informed consent, no prior diagnosis of liver cirrhosis, minimum age of 35 years, eligible for health check‐up [every 2 years, since April 2019 every 3 years]) were screened for cirrhosis and fibrosis. The screening procedure itself consisted of the additional determination of two serum surrogate markers and the calculation of the aspartate aminotransferase to platelet ratio index (APRI) in primary care (first stage).

For the SEAL programme, a cut‐off value of 0.5 was chosen. In the case of a higher APRI in combination with at least one pathological transaminase, patients were considered positively screened patients with conspicuous liver values. Thus, a positive screening rate of 3.5%–4.0% was expected for the SEAL cohort with a false‐positive screening rate at the first stage of 70%–80%.^23^, ^24^ Positively screened patients were referred to gastroenterological specialist examination for further clarification (second and third stage).

Within the SEAL programme, the present study was designed to explore potential psychosocial consequences, as well as behavioural changes for positively screened patients within the screening process. Since these patterns are complex and highly individual, a qualitative approach represents the best design to systematically explore all potential reactions and processes related to liver screening within the subjective reality of the concerned. Furthermore, with this study, we want to focus on the patient's perspective, which is often given too little attention when implementing new medical interventions. The guiding research questions are as follows:

1. Are there negative psychosocial consequences in relation to the screening?
2. Which factors are related to psychosocial consequences?
3. Are there (behavioural) reactions in relation to the screening to cope with psychosocial consequences?
4. What are the attitudes towards screening after receiving a (false) positive test result, in general and specifically towards the SEAL programme?

## 2 MATERIALS AND METHODS

The methods and results in this article are reported using the consolidated criteria for reporting qualitative research (COREQ) checklist.^25^

### 2.1 Participants and procedures

To identify positively screened patients, an interface to the electronic case report form (eCRF) was installed. The eCRF served as a management tool within the SEAL programme to collect all relevant patient data. When a new referral of a patient to a specialist was documented in the eCRF, the study team received an e‐mail information. Immediately afterwards, the patient was contacted by the first author via mail including a patient information and informed consent. He approached the interviewees with this information, including a statement that the study is independent of their medical treatment. The researchers were not in contact with the physicians or clinics of the patients and had no relationship before the study commencement. Incentives of €50 (in form of a transfer to a private account after the interview) were used to increase the response rate for this study since we received no responses within the first 4 months of recruiting. All patients, who were positively screened in the SEAL programme in the period between June 2018 and May 2019 were asked to take part in the study (*n* = 158). With a response rate of 7%, we could realise *n* = 11 telephone interviews. Initially, a purposeful sampling strategy based on the criteria sex, age, comorbidity level and federal state was planned. However, since the recruiting phase took more than 1 year and the response rate was unexpected low, recruiting stopped after reaching interviews with *n* = 11 patients. The resulting convenience sample was based on the researchers' considerations that this sample size is (a) sufficient to reach data saturation and (b) feasible to analyse within the available resources. This rationale is supported by a work of Guest et al.^26^ concluding that most themes emerge after 6–12 interviews. In some cases, a recall appointment (second stage) at the specialist was already scheduled at the time of the interview, hence a second talk after the consultation could be realised (*n* = 4). This option was offered to include further patient experiences even after correction or confirmation of a preliminary screening result to receive a more holistic impression. To keep the recruiting period in limits, we did not offer this for patients, who had no follow‐up consultation scheduled.

To explore short‐term psychosocial consequences homogeneously, we tried to realise the interviews as soon as possible after the initial mail approach. However, due to delays in communication and documentation in the eCRF, the time between physician visit and realisation of each interview varied.

For data collection, an interview guideline was developed based on the research questions. The guidelines covered four main topics: information about the screening, reactions to the results, external information retrieval and attitudes towards screening (see Supporting Information Material). This guideline was consented within a team of field experts (psychology, sociology, health services research and gastroenterology) and was pilot tested in the first interview. During the interviews, field notes were made to complement the data.

The telephone interviews consisted of two parts. First, an open narrative part, semi‐structured by the guidelines, covered the main topics enabling the patients to speak openly. The second part was a short standardised query of sociodemographics, for example, age, education and comorbidities.

### 2.2 Interview setting

All interviews were carried out by the first author (male), who has extensive experience in both qualitative and quantitative methods. He holds a Master of Science in Sociology and Empirical Social Sciences and was occupied as a research assistant at the University Hospital of Freiburg at the time of the interviews. At the beginning of each interview, the interviewer introduced himself, explained the goal of this study and repeated key information that was presented in the patient information sent out in advance. The interviewer emphasised that he has no medical profession and that this study is not related in any way to the medical treatment of the patients. All interviews were conducted by telephone while the respondents were at home. No presence of other cohabitants interfered with the interviews. Transcripts and findings were not returned to participants for comment and or correction; however, the respondents were encouraged to contact the researcher after the interview in case of any upcoming thoughts or supplementary requests as a consequence of the interview.

### 2.3 Data analysis

In sum, audio material of 4.5 h (approximately 19 min/call) was recorded. Audio data were transcribed verbatim by an external service provider and have been checked twice to ensure accuracy. The analysis was conducted by the first author and followed the structuring content analysis approach by Kuckartz^27^ using MAXQDA PLUS 2020 software. Thereby, categories were first defined deductively based on the research questions and assigned as part of the first coding procedure. Second, the categories were revised inductively based on the data, subcategories were formed, and a subsequent coding procedure was applied. When topics were addressed multiple times, they were also coded multiple times as text passages. The coding tree is available as Supporting Information Material.

## 3 RESULTS

### 3.1 Analysis sample

In sum, nine women and two men were interviewed (see Table 1). One person was accidentally included in the SEAL programme, though she did not fit the inclusion criteria for the main study (35 years or older). However, since this was not an exclusion criteria for the qualitative study, we decided to keep the interview data in the sense of a holistic approach. The mean age of the respondents was 65 years, however, since the mean is biased by the minimum extreme of 30 years, the median age (72 years) provides a better impression. In comparison to the general SEAL population, our sample was, on average, 12 years older (median age of the whole SEAL population was 60 years) and less balanced regarding sex (SEAL population: 54.5% women). On average, three pre‐existing conditions were mentioned with a minimum of one and a maximum of six. The majority of our sample reported to have low (54.5%) education (see Table 1) which is assumed to be associated with low health literacy.^28^, ^29^

**Table 1:** Sample characteristics.

Sex	Age	Comorbidity/pre‐existing conditions	Education
F	72	Neurological disorder	Low
M	55	Hypertension, respiratory organ disease, musculoskeletal disease, cancer	Low
F	72	Hypertension, liver/gall bladder disease, musculoskeletal disease, dejection	Medium
F	72	Hypertension, respiratory organ disease, liver/gall bladder disease, diabetes	Medium
F	74	Hypertension, circulatory/vascular disease, gastrointestinal disease, diabetes, neurological disorder	Medium
M	66	Heart attack, diabetes	Low
F	30	Respiratory organ disease, gastrointestinal disease	Low
F	59	Hypertension, kidney disease, musculoskeletal disease, cancer, dejection/anxiety	Medium
F	62	Hypertension, liver/gall bladder disease	High
F	77	Hypertension, circulatory/vascular disease, gastrointestinal disease, diabetes, musculoskeletal disease, cancer	Low
F	78	Heart attack	Low

Abbreviations: F, female; High, university‐entrance diploma/vocational diploma; Low, no certificate or elementary/secondary school leaving certificate; M, male; Medium, general certificate of secondary education.

The following four sections refer to the four research questions step by step.

### 3.2 Perceived psychosocial consequences

Overall, the short‐term negative consequences of screening were limited. With the exception of one patient (P 5), who reported that her liver values were good, each patient commented on their own emotions regarding excessively high liver values (see Table 2). Those were differentiated into negative and positive emotions, represented by two main categories.

**Table 2:** Reported emotions on liver screening results.

Patient	Pos (+)/neg (−)	Patient statement to the question: ‘How do/did you feel with the screening result?’
1	+	‘Normal, like normal, I'm quite honest. […] And I also feel comfortable and… that's why’.
2	+	‘I didn't really worry about the liver. […] I'm fine, perfectly happy’.
3	−	‘If there is still said (laughs), the values are so bad, if the doctor is already afraid, then you also get scared’.
4	−	‘Oh God, then I say: O.k., then it's like this. I've lived my life, done, then it's just over. So let's put it this way, I don't have a strong will to live right now’.
5	/	/
6	+	‘No, directly worried not. […] I thought, oh well, if he means in ten years, then it's not so bad (laughs)’.
7	+	‘No, I mean, I can't do more than pay attention anyway’.
8	−	‘So emotionally I was in a bad way, until they told me yesterday that I… that it is not liver cancer. […] Sometimes you think, well, it won't be anything, and other times you get carried away and think that you have more’.
9	−	‘I was already a bit… […] I thought: wait and see what he says first, but of course I was relieved when he then wrote: no signs’.
10	+	‘I'm not one to be afraid of anything. I think it's all good. […] Deep down, I knew it and now I've had that confirmed, and now I'm happy’.
11	−	‘So not so good. I was thinking, first the heart, now the liver is coming too’.

In sum, five patients reported negative emotions about the screening results and five patients reported positive or at least neutral emotions in relation to the screening results. The negative emotions can be described as fear and sorrow as well as resignation (P 3, 8 and 11). In one case, the emotion was expressed as reduced will to live (P 4). However, the latter should not be attributed solely to screening since the person explained other circumstances that altogether led to this state (e.g., recent death of a family member).

The positive emotions were characterised by comfort (P 1), happiness (P 2 and 10) and self‐care (P 7). One patient used humour to answer the question (P 6).

### 3.3 Factors related to psychosocial consequences

Two patients explained in a follow‐up interview when the screening results turned out to be false positive that their negative emotions were blown away after conclusive findings. This result points to the uncertainty periods during the waiting time between a preliminary screening result and the confirmatory/corrective diagnosis. On the one hand, time under uncertainty plays an important role with regard to the manifestation of negative emotions. On the other hand, functional patient–provider communication is the key to reduce the burden of uncertainty. Every interviewee who described negative emotions also reported about suboptimal physician–patient interaction including a lack of trust, regardless of whether the screening result was verified or not:

*Oh well, I don't have any confidence.* (P 3)

*Nobody asked me about that. […] I have to go back there now, and then I will also address this. That's why I say I'm annoyed with myself that I didn't address it right away.* (P 8)

*I have to get myself very involved. Very strongly.* (P 11)

On the other hand, patients who showed no negative emotions emphasised the good relationship to their health care providers, which were untouched by false‐positive results:

*I trust my doctor blindly.* (P 2)

*He's good. […] It's the family doctor. You only go to him if you are satisfied.* (P 5)

*Very great trust.* (P 7)

It seems that a good information flow with transparent communication of comprehensive health information builds trustworthy patient‐provider relationships and reduces the risk of developing negative emotions in the context of screening. Here, it is important that the educational level and cognitive capabilities, respectively, health literacy of the patients are taken into account.

*I don't understand medicine at all*. (P 3)

*He told me that, but I couldn't remember it because, yes, there are always such special names*. (P 7)

### 3.4 Behavioural reactions and coping

Patients were asked how they reacted to upcoming negative emotions or insufficient information concerning their screening results. The majority of the interviewees reported that they have searched for external information sources and resources of social support. Both were most often found in friends or acquaintances who were described as having general knowledge in the field of medicine.

*This is my godchild, she's an occupational therapist: tell me what the liver values are, please. And she told me, then I knew*. (P 1)

*The pharmacist also told me not to worry, it wouldn't be that bad*. (P 1)

*I have a niece who works at the pharmacy. I thought when she came she would look at it (groans), but so far she hasn't come*. (P 3)

*My brother was a geriatric nurse, and he also had a bit of an idea. […] And then we talk about it from time to time*. (P 6)

This result once again emphasises the need for a well‐working patient–provider communication, since otherwise external information sources that might not be qualified are consulted to reduce the information gap.

### 3.5 Future screening attitudes

Overall, the majority of respondents were positive about screening procedures and especially about the SEAL programme. It was striking that even three of the participants who reported negative emotions during the screening process were clearly in favour of the screening programme. The decisive factor was a smooth and fast diagnosis process.

*Yes, that's reassuring. So, I have to say, that was the best thing I could have done, to agree to the project here, because I do notice that it goes hand in hand, it goes quickly, and you're in good hands*. (P 8)

*After all, it doesn't hurt. And it's reassuring to know that nothing will change for the worse*. (P 9)

*And then that result afterwards with the better knowledge […] is absolutely an advantage in any case*. (P 10)

*Yes, I think that's a good thing, if you're in treatment there and you're being questioned. And, if it's nothing, it's also good*. (P 11)

## 4 DISCUSSION

This study explored the experiences of patients who were screened (false) positive for cirrhosis or advanced fibrosis in Germany, including four patients who received a confirmatory diagnosis about hepatic liver injury present.

The results partly fit with previous work, as only some respondents described negative emotional consequences related to the screening itself. The latter seems to be mostly driven by insufficient informational support, which plays a key role in dealing with diagnoses, especially when health literacy is low.^30^, ^31^, ^32^, ^33^ Additionally, negative psychosocial consequences of screening might be worsened, when suboptimal patient–provider communication impedes the transfer of transparent information.^34^ As a result, patients tend to ask for information in a low‐threshold area, that is, their social environment. Even though the respondents reported that their social supporters were considered as having medical knowledge, they are no experts in gastrointestinal screening. Therefore, this informational support seeking behaviour might, on the one hand, have beneficial effects (e.g., bringing relief), but on the other hand, it might also carry a risk of misleading information and thus should be avoided by optimising the information flow and by strengthening confidence in the patient–provider relationship.^35^

The limitations of this study are that, due to unclear documentation and communication delays, it was not possible to control the duration between the screening of the participants and the interview. We have to assume that there is an indeterminable time offset between the check‐up, the documentation in the eCRF and the initial approach, respectively the realisation of the interviews. Thus, recall bias might have occurred. Additionally, this leads to an unclear sample of participants who report about very recent reactions and individuals with larger narrative periods. Further bias in our results might be caused due to the low response rate of the sample (7%). A possible explanation for this could be that some participants were not aware of the name of the screening programme and were confused by our affiliation (Freiburg) since the programme was conducted in other Federal States of Germany.

Since we could not follow our initially planned purposeful sampling approach, we could not entirely influence the composition of our sample so that patients with higher education, as well as men, were underrepresented. As a consequence, we could also not explicitly integrate the views of vulnerable groups.^36^

In terms of sampling, a certain selectivity might have occurred, since patients with high psychosocial load might be not in the state to participate in a study.

Furthermore, it is important to state that this study does not focus on long‐term psychosocial consequences and it is not clear whether some of the reported emotions persist or aggravate over time.

From a methodological point of view, face‐to‐face interviews might have gained deeper information about the situation of the patients. In telephone interviews, the information about the interlocutor is limited because no visual stimuli (e.g., gestures) are present to improve the relationship between the interviewer and the interviewee.

## 5 CONCLUSION

To reduce the potential occurrence of negative psychosocial consequences during the screening process, medical screening should always be performed in the context of well‐communicated and transparent information. Our results emphasise that measures to improve health communication on the side of health professionals (organisational component) and measures to increase health literacy on the side of patients (individual component) might contribute to avoid negative emotions in line with the screening. This result also illustrates how important it is to foster health literacy from a public health perspective. Here, future studies from other countries might contribute to an international comparison of our results. In addition, our data indicate that the waiting time for clarification was perceived to be stressful. Thus, a smooth and fast diagnosis process not only may contribute to an overall positive attitude towards screening but also reduces burdensome periods.

This study revealed a key role of health communication for the evolution of negative emotions in relation to screening. Since health communication is a major problem in the context of health care for vulnerable groups, it is important to integrate the view of migrants, low‐educated people and patients with cognitive deficits in further research on this topic.

## AUTHOR CONTRIBUTIONS

Urs A. Fichtner and Erik Farin‐Glattacker developed the interview guidelines, interviewed the patients, coded and analysed the data. Urs A. Fichtner drafted the paper. All authors contributed to subsequent drafts and the final version.

## CONFLICT OF INTEREST STATEMENT

The authors declare no conflict of interest.

## ETHICS STATEMENT

This substudy of the SEAL programme was performed in line with the principles of the Declaration of Helsinki. The substudy has been granted by the Ethics Committees of Rhineland‐Palatinate and Saarland (reference number 837.361.17 [11195]). Furthermore, it was reviewed and approved by the data security officer of the Medical Center of the University of Freiburg. The SEAL programme was registered at the German Registry for Clinical Studies (DRKS) under the ID DRKS00013460. Data collection was based on written informed consent. For this purpose, we sent all respondents information material and a consent form via mail before the telephone interviews. Consent for publication is not applicable.

## Supporting information

Supporting information.

Supporting information.

## Data Availability

Due to ethical considerations and German data protection law, the individual patient data cannot be published.
