# Supplementary material for: How do (false) positively screened patients experience a screening programme for liver cirrhosis or fibrosis in Germany? A qualitative study
Source: Health Expect. 2023 Jun 19;26(5):1923–30. doi: 10.1111/hex.13800 (PMC10485303; doi:10.1111/hex.13800)
Supplement: Supplementary file 1 — Supporting information. [file HEX-26--s001.docx]

**Coding Tree**

| Main category | Subcategories |
| --- | --- |
| Psychosocial consequences |  |
|  | Positive |
|  | Negative |
|  | Emotional |
|  | Behavioral |
| Patient-provider relationship |  |
|  | Active |
|  | Passive |
|  | Positive characteristics |
|  | Negative characteristics |
|  | Trust / competence |
| Information flow |  |
|  | Awareness of study/programme participation |
|  | Comprehension problems / health literacy |
|  | Use of external information sources |
| Barriers in the screening programme |  |
|  | (Waiting) time |
|  | Distance |
| Attitudes towards screening |  |
|  | Knowledge of other screenings |
|  | Positive attitudes |
|  | Negative attitudes |
