# Supplementary material for: How do (false) positively screened patients experience a screening programme for liver cirrhosis or fibrosis in Germany? A qualitative study
Source: Health Expect. 2023 Jun 19;26(5):1923–30. doi: 10.1111/hex.13800 (PMC10485303; doi:10.1111/hex.13800)
Supplement: Supplementary file 2 — Supporting information. [file HEX-26--s002.docx]

**Guideline patient interviews t_1_**

**Preparation of the Interview:**

| **Contact** |
| --- |
| - Introduction: - Profession, research associate at Freiburg University Hospital, influence on treatment. - Topic: - We are interested in the experiences of patients who participated in Checkup 35 as part of the SEAL (liver screening) study. Your personal experiences with the treatment process are important to us. - Procedure: - This form of interview is primarily about you telling me your story. There is no right or wrong there. I'll ask you a question now and then or give you a few bullet points, and you just tell me what comes to mind. We have half an hour for the interview. |
| **Data security** |
| Data protection is important to us, so I would like to briefly go over a few things with you again:   - We had sent you a letter with a participation explanation and consent form. You signed this and sent it back to us. - The interview we are conducting together today will be audio-digitally recorded and transcribed at a later date. - Names, locations and any details that would allow us to infer who you are will be anonymized and kept strictly confidential. - Personal contact details will be kept separately and destroyed at the end of the project. - The data obtained will be used exclusively for research purposes. |
| **Introduction** |
| I'll turn on the tape recorder now, and then I'll ask you the first question. |

| **Question 1** |
| --- |
| As part of the SEAL liver screening program, various values are determined during the checkup. One of them is a liver value. Have you been told your liver value?  Follow up:   - How was the value communicated? - Do you know how this value is called? - What were you told about the importance of this value? - Has your family doctor told you anything about the risks and consequences of an elevated value? |
| **Question 2** |
| How did you react on those news?  Follow up:   - How are you doing emotionally with this? Are you worried about it? Change from before? - If worried, have you talked to friends or family about these concerns? - What thoughts did you have after sharing this value? - Have you specifically identified things in your life that you would like to change to lower your liver score? - Have you specifically done things in response to this value? |
| **Question 3** |
| After being told about this value, did you learn about the significance of the liver value in any other way outside of the doctor's office?  Follow up:   - For example, did you see another doctor, research information on the Internet, contact friends or family? - What did you find out? - How did you respond to this other information? - Emotionally, Behaviorally, Risk Factors? |
| **Question 4** |
| Now I would like to ask you some questions about your attitudes:  What is your attitude towards screening procedures?   - Understanding of terms? - Generally and explicitly this liver screening   Has your attitude towards screening procedures changed in the last few weeks?   - Why?   In general, do you have confidence in what doctors tell you?   - Do you have confidence in your primary care physician? - Please specify. What do you base that on? |

That brings us to the end of the interview.

May I ask you what the next steps are for you medically? Have you already made an appointment with a specialist or at the liver center?

| **□** | **□** |
| --- | --- |
| Specialist | Liver center |

Date on: ______________ in (time frame) _______________

Do you agree to me contacting you again after the appointment in a few weeks?

| **□** | **□** |
| --- | --- |
| Yes | No |

**Guideline patient interviews t_2_**

| **Question 1** |
| --- |
| Our last telephone conversation took place after you had been to your family doctor and your liver value had been determined. How did you feel after our last phone call?  Follow up:   - Mental? How did you feel about your health? - Were you scared/worried? Were you nervous about further clarification? - How did you behave? Did you seek additional information? Did you contact other doctors? Internet? Other sources of information? - Did you talk about it with friends/family? - Have you changed your health behaviors? |
| **Question 2** |
| Since then, you have had another appointment with a specialist (or liver center). What were you told there?  Follow up:   - What finding? How were you told about this finding? - What were you told about the significance of this finding? - How did you react to this finding? - How have you felt since the finding? Anxiety? Worried? - Were you told about any risk factors related to your liver health? - Were you given any recommendations for action regarding your liver health? - Have you done anything specifically in response to this finding? |
| **Question 3** |
| After being told of this finding, did you learn about the significance of the liver value in any other way outside of the physician's office?  Follow up:   - For example, did you see another doctor, research information on the Internet, contact friends or family? - What did you find out? - How did you respond to this other information? - Emotionally, Behaviorally, Risk Factors |
| **Question 4** |
| Now I would like to ask you some questions about your attitudes:  What is your attitude towards screening procedures?   - Understanding of terms? - Generally and explicitly this liver screening   Has your attitude towards screening procedures changed in the last few weeks?   - Why?   In general, do you have confidence in what doctors tell you?   - Do you have confidence in your specialist / liver center? Please specify. What do you base that on? |
